# Unravelling the role of *PLK1* in tumorigenesis by revealing the mutational landscape of colorectal and lung cancer with *PLK1* mutations

**DOI:** 10.1111/jcmm.18497

**Published:** 2024-06-18

**Authors:** Shuo Wang, Feng Gao, Yinghui Bi, Xiaotian Zhao, Qiuxiang Ou, Minyi Zhu, Xue Wu, Xuefei Zhang, Kaiping Mao

**Affiliations:** ^1^ Key laboratory of Carcinogenesis and Translational Research (Ministry of Education), Urological Department Peking University Cancer Hospital & Institute Beijing China; ^2^ Department of Medical Oncology Beidahuang Industry Group General Hospital Harbin China; ^3^ Department of Oncology Qingdao Hospital, University of Health and Rehabilitation Sciences (Qingdao Municipal Hospital) Qingdao China; ^4^ Geneseeq Research Institute Nanjing Geneseeq Technology Inc. Nanjing China; ^5^ Department of Thoracic Surgery I The Second Hospital of Dalian Medical University Dalian China; ^6^ Department of Thoracic Surgery The Affiliated Hospital of Qingdao University Qingdao China

**Keywords:** colorectal cancer, lung cancer, mutational landscape, *PLK1*, tumour‐suppressor feature

Polo‐like kinase 1 (PLK1), encoded by the *PLK1* gene and mainly consisting of a Pkinase domain and two Polo‐box domains (PB1 and PB2),^1^ is generally considered as a cancer promoter, owing to its critical role in cell cycle and overexpression.^2^ Recent studies have revealed that *PLK1* might play as a tumour‐suppressor gene,^3^, ^4^ and limited preclinical achievements of PLK1 inhibitors have been translated into good clinical outcomes.^5^ Considering concurrent driver and passenger mutations involved in developing treatment plan, genomic profiles are critical to *PLK1*‐mutated patients receiving PLK1 inhibitor combination therapy.

Herein, of 398 *PLK1*‐mutated pan‐cancer tissue samples [including lung cancer, colorectal cancer (CRC), breast cancer, hepatobiliary cancer, genitourinary cancer, uterine cancer, etc.], we enrolled 126 patients with lung cancer (the LC cohort) and 160 patients with CRC (the CRC cohort) to profile the genomic landscape of *PLK1* and concomitant mutations under natural selection within lung cancer and CRC, detailed in Table S1 and Table S2, respectively (Figure 1A). In addition to comparing the genomic features of the CRC cohort to 160 *PLK1* wild‐type CRCs, further investigation between 63 *PLK1*‐mutated (the cBioPortal CRC cohort) and wild‐type CRCs in the cBioPortal database was performed to study the role of *PLK1* in CRC development (See Appendix S1 for methods).

**FIGURE 1:**
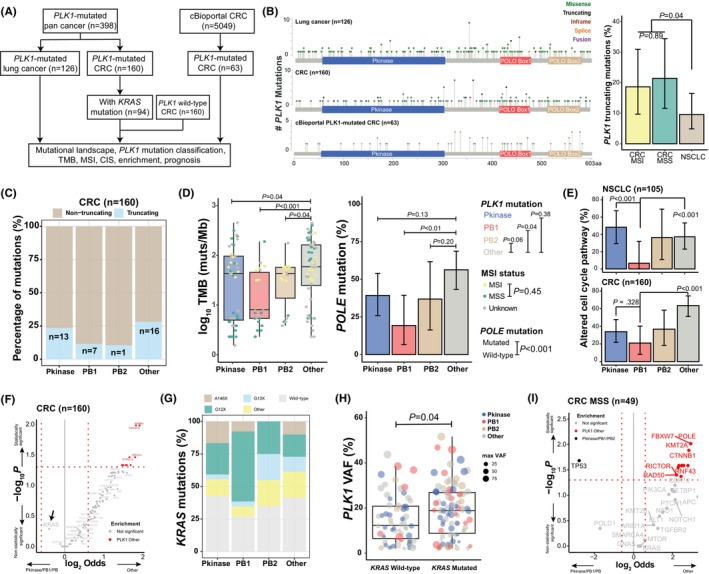
Patient inclusion and molecular features related to *PLK1* mutation subtype. (A) The flow chart of lung cancer and colorectal cancer (CRC) patient inclusion, including 126 *PLK1*‐mutated lung cancer patients, 160 CRCs and 160 *PLK1* wild‐type CRCs from the Nanjing Geneseeq Technology Inc. database, and CRCs from the cBioportal database. (B) *PLK1* mutations at multiple gene regions were identified, including Pkinase mutations (the blue region), PB1 mutations (the red region), PB2 mutations (the brown region) and Other mutations (the grey region), in the LC, CRC cohort and cBioPortal CRC cohorts. No obvious hotspots were observed, and *PLK1* truncating mutations were more common in microsatellite instable (MSI) and microsatellite stable (MSS) CRC than in non‐small cell lung cancer (NSCLC). (C) The detection rates of *PLK1* truncating mutations of each subgroup in the CRC cohort. (D) The tumour mutation burden (TMB) of each subgroup of NSCLCs in the LC cohort and the CRC cohort. (E) The prevalence of mutated cell cycle pathway in *PLK1*‐mutated NSCLCs and CRCs. (F) The enrichment of mutated genes between CRC with *PLK1* Other mutations and *PLK1* Pkinase/PB1/PB2 mutations. (G) The detection rate of *KRAS* mutations of each subgroup in the CRC cohort, with more *KRAS* ^*G12X*^ mutations detected in the PB1 subgroup than the Other subgroup. (H) *PLK1* mutation variant allele frequencies (VAF) were higher in *KRAS*‐mutated CRC patients than in *KRAS* wild‐type CRC. (I) The enrichment of mutated genes between MSS CRC with *PLK1* Other mutations and *PLK1* Pkinase/PB1/PB2 mutations.

The mutational landscapes and clinical characteristics of the CRC and LC cohorts were summarized in Figure S1A. The CRC cohort included 45 microsatellite instable (MSI) CRCs, 49 microsatellite stable (MSS) CRCs and 66 CRCs with unknown MSI status. *TP53*, *APC*, *KRAS*, *KMT2B* and *PIK3CA* were frequently mutated in CRCs harbouring *PLK1* mutations. The LC cohort consisted of 105 patients with non‐small cell lung cancer (NSCLC), three patients with small cell lung cancer and 18 lung cancer patients with unknown histological subtypes. Similar to the CRC cohort, *TP53*, *KRAS* and *EGFR* mutations were relatively common in the LC cohort, whereas *APC*, *KMT2B* and *PIK3CA* were less frequently altered.

*PLK1* mutation subtypes in the LC, CRC and cBioPortal CRC cohorts were summarized in Figure 1B. According to the genomic region, *PLK1* mutations were categorized into the Pkinase (blue), PB1 (red), PB2 (brown) or other (grey) subgroup. Of note, neither genomic aberrations leading to amino acid substitutions at the position 131 of *PLK1* (*PLK1* ^*E131X*^) nor those at position 133 (*PLK1* ^*C133X*^), which were related to the direct interaction between PLK1 inhibitors and the Pkinase domain,^1^ were detected in three study cohorts. Besides considerable *PLK1* missense mutations, various truncating mutations were observed, especially in CRC, in which truncating mutations were more common than in lung cancer (*p* = 0.02, Figure S1B). Moreover, the prevalence of *PLK1* truncating mutations was comparable between MSI and MSS CRCs (*p* = 0.89), which was higher than that of NSCLC (*p* = 0.04, Figure 1B). However, we were unable to identify obvious *PLK1* hotspots under natural selection in the CRC or LC cohorts, which is non‐intuitive if *PLK1* plays as an oncogene. In the CRC cohort, compared to CRC harbouring PB1/2 mutations, *PLK1* truncating mutations appeared to be more prevalent in the Pkinase domains or the regions other than Pkinase and PB1/2 (Other) (*p* = 0.05, Figure 1C), with no obvious differences between MSI and MSS CRCs (Figure S1C). By contrast, this trend could not be detected in NSCLCs of the LC cohort.

*PLK1*‐mutated CRCs exhibited higher tumour mutation burden (TMB) than *PLK1*‐mutated NSCLCs, which were the majority of the LC cohort (Figure S1D). In the CRC cohort, TMB appeared to be higher in patients harbouring *PLK1* Other mutations than in those with *PLK1* Pkinase (*p* = 0.04), PB1 (*p* < 0.001) or PB2 (*p* = 0.04) mutations (Figure 1D). Intriguingly, the difference in prevalence of *POLE* somatic mutations was similar to the TMB levels across four subgroups (Figure 1D). The results of a multivariable linear regression model adjusting for MSI and *POLE* mutational status demonstrated relatively high TMB in the Other subgroup (vs. PB1, *p* = 0.04; vs. PB2, *p* = 0.06) and no significant TMB difference between MSI and MSS CRCs (*p* = 0.45, Figure 1D). Additionally, *PLK1* Other mutations might be associated with higher prevalence of mutated cell cycle pathway in both NSCLC and CRC, particularly in comparison to *PLK1* PB1 mutations (*p* < 0.001, Figure 1E). Consistent with our findings in lung cancer TMB, there were no mutations obviously enriched in lung cancers with *PLK1* Other mutations (Figure S1E), whereas considerable mutated genes were more common in CRCs with *PLK1* Other mutations (Figure 1F). Interestingly, mutations leading to amino acid substitutions at the position 12 of *KRAS* (*KRAS* ^*G12X*^) were more prevalent in CRCs carrying *PLK1* PB1 mutations (*p* < 0.001, Figure 1G), suggesting a potential cooccurrence of *KRAS* dysfunction and PLK1 PB1 domain abnormalities in CRC. *PLK1*‐mutated CRCs with concurrent *KRAS* mutations had higher *PLK1* mutation variant allele frequencies than those without (*p* = 0.03, Figure 1H). Additionally, *TP53* mutations exhibiting cooccurrence with *PLK1* mutations in Pkinase, PB1 or PB2 regions were exclusively observed in MSS CRCs while multiple genetic alterations were enriched in MSS CRCs with *PLK1* Other mutations (Figure 1I).

As our findings suggested a stronger association of *PLK1* mutations with concomitant mutations in CRC than in lung cancer, we further performed comparison of other genomic features between *PLK1*‐mutated and wild‐type CRCs. In the cBioPortal database, MSI was more prevalent in *PLK1*‐mutated CRCs than in those with wild‐type counterparts (*p* < 0.001, Figure 2A). Compared to *PLK1* wild‐type CRCs, the CRC cohort showed higher TMB (*p* < 0.001, Figure 2B) whereas similar TMB levels were observed in CRCs with *PLK1* truncating and non‐truncating mutations (*p* = 0.34), suggesting that more genetic abnormalities were accumulated in *PLK1*‐mutated CRCs without obvious differences between truncating and non‐truncating mutations. Intriguingly, the prevalence of *APC* and *KRAS* mutations, which are well‐known CRC drivers, was comparable between CRCs with and without *PLK1* mutations; however, *TP53* mutations were more prevalent in *PLK1* wild‐type CRCs while considerable mutated genes were enriched in *PLK1*‐mutated CRCs (Figure 2C). Moreover, *TP53* mutations were common in CRCs with non‐truncating *PLK1* Other mutations than those with truncating *PLK1* Other mutations (*p* = 0.01, Figure 2D). We supposed that non‐truncating *PLK1* mutations in non‐functional domains might have limited influence on CRC development, requiring more involvement of *TP53* dysfunction. Furthermore, relatively high chromosome instability scores were observed in *PLK1* wild‐type CRCs in comparison to CRCs harbouring *PLK1* non‐truncating mutations (*p* = 0.07, Figure 2E) when the confounding effect of MSI (*p* < 0.001) and *POLE* (*p* = 0.03) mutational status were controlled for; however, the difference between CRCs with *PLK1* truncating mutations and with wild‐type *PLK1* was no longer significant (*p* = 0.37).

**FIGURE 2:**
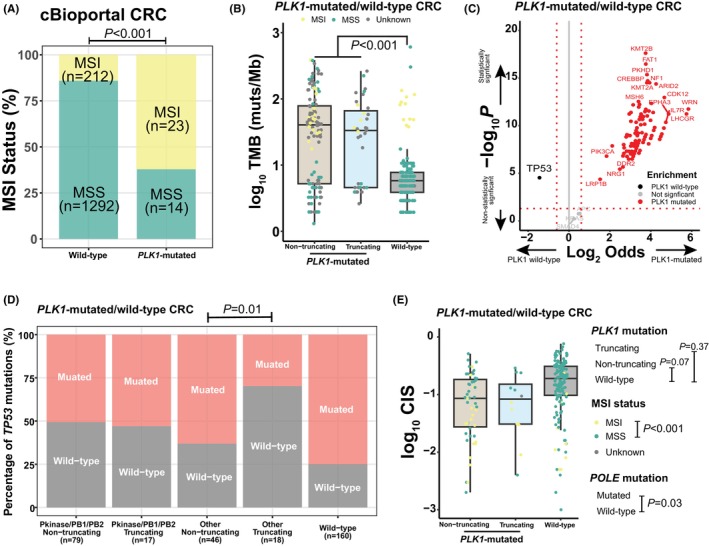
The role of *PLK1* in tumorigenesis under natural selection within CRC development. (A) In the cBioPortal database, microsatellites instable (MSI) colorectal cancer (CRC) were more frequently observed in *PLK1*‐mutated patients than in *PLK1* wild‐type patients. (B) The tumour mutation burden (TMB) level of *PLK1* wild‐type samples was lower than that of samples detected with *PLK1* truncating or non‐truncating mutations. (C) *TP53* mutations were enriched in *PLK1* wild‐type samples when compared to *PLK1*‐mutated CRCs, while considerable mutated genes were more prevalent in *PLK1*‐mutated CRCs. (D) *TP53* mutations appeared to be more common in CRC with non‐truncating *PLK1* Other mutations than CRC with truncating *PLK1* Other mutations. (E) Chromosome instability scores (CIS) were higher in *PLK1* wild‐type CRC than CRC with *PLK1* non‐truncating CRC.

To summarize, based on the genomic landscape under natural selection within lung cancer and CRC tumours, the clinical application of PLK1 inhibitors still needs to be approached with caution given potent tumour‐suppressor features of *PLK1*. Our study had some limitations that need to be considered. First, most patients enrolled in this study had missing information about cancer stage, treatment and prognosis data, which restricted the study of the association between *PLK1* mutation subtypes and treatment efficacy. Larger *PLK1*‐mutated CRC and lung cancer populations are also required to explore the interaction between *PLK1* and *KRAS* mutations in CRC and compare the molecular features between *PLK1*‐mutated NSCLC and small cell lung cancer, respectively. In addition, our findings were mainly based on CRC and NSCLC samples, and further studies focused on other cancer types are needed. Finally, future experimental investigation and validation are warranted to functionally determine whether *PLK1* mutations would affect PLK1 inhibitor binding.

## AUTHOR CONTRIBUTIONS

**Shuo Wang:** Data curation (lead); formal analysis (equal); funding acquisition (equal); investigation (equal); validation (equal); writing – original draft (lead); writing – review and editing (equal). **Feng Gao:** Data curation (lead); formal analysis (equal); investigation (equal); resources (equal); validation (equal); writing – original draft (lead); writing – review and editing (equal). **Yinghui Bi:** Data curation (equal); formal analysis (equal); investigation (equal); writing – original draft (equal); writing – review and editing (equal). **Xiaotian Zhao:** Formal analysis (equal); investigation (equal); methodology (equal); software (equal); visualization (equal); writing – original draft (equal); writing – review and editing (equal). **Qiuxiang Ou:** Formal analysis (equal); investigation (equal); project administration (supporting); visualization (equal); writing – original draft (equal); writing – review and editing (equal). **Minyi Zhu:** Formal analysis (equal); investigation (equal); visualization (equal); writing – original draft (equal); writing – review and editing (equal). **Xue Wu:** Formal analysis (equal); investigation (equal); project administration (supporting); supervision (supporting); writing – original draft (equal); writing – review and editing (equal). **Xuefei Zhang:** Conceptualization (equal); funding acquisition (equal); investigation (supporting); project administration (equal); resources (equal); supervision (lead); writing – original draft (equal); writing – review and editing (equal). **Kaiping Mao:** Conceptualization (equal); methodology (equal); project administration (equal); resources (equal); supervision (lead); writing – original draft (equal); writing – review and editing (equal).

## FUNDING INFORMATION

This study was supported by the Science foundation of Peking University Cancer Hospital (No. 2021‐7, to Shuo Wang), the Special Fund for Clinical Research of Wu Jieping Medical Foundation (No. 320.6750.2021‐16‐8, to Xuefei Zhang), and ‘1 + X’ program for Clinical Competency Enhancement‐Clinical Research Incubation Project, the Second Hospital of Dalian Medical University (No. 2022LCYJYB09, to Xuefei Zhang).

## CONFLICT OF INTEREST STATEMENT

Xiaotian Zhao, Qiuxiang Ou, Minyi Zhu, and Xue Wu are employees of Nanjing Geneseeq Technology Inc., China. The remaining authors have nothing to disclose.

## Supporting information


Figure S1.

Table S1.

Table S2.

Appendix S1.

## Data Availability

The mutation list of 286 tissue samples is provided as the Table S2, and other datasets used and/or analysed during the current study are available from the corresponding author on reasonable request. The external combined CRC dataset was obtained from the cBioPortal database (https://bit.ly/43D5zsm).
