# Supplementary material for: Unravelling the role of PLK1 in tumorigenesis by revealing the mutational landscape of colorectal and lung cancer with PLK1 mutations
Source: J Cell Mol Med. 2024 Jun 18;28(12):e18497. doi: 10.1111/jcmm.18497 (PMC11184281; doi:10.1111/jcmm.18497)
Supplement: Supplementary file 1 — Figure S1. [file JCMM-28-e18497-s001.zip › Figure S1.docx]

Figure S1: (A) The mutational landscapes of included colorectal cancer (CRC) and lung cancer samples. (B) The proportion of *PLK1* truncating mutations in CRC, lung cancer, and cancers other than CRC and lung cancer. (C) The proportion of *PLK1* truncating mutations in each subgroup of CRCs with different microsatellites instable (MSI) status. (D) The tumour mutation burden (TMB) of each subgroup of NSCLCs and CRCs. (E) The enrichment of mutated genes in lung cancer with *PLK1* Pkinase/PB1/PB2 mutations in comparison to lung cancer with *PLK1* Other mutations.
