# Supplementary figures and images for: Unravelling the role of PLK1 in tumorigenesis by revealing the mutational landscape of colorectal and lung cancer with PLK1 mutations
Source: J Cell Mol Med. 2024 Jun 18;28(12):e18497. doi: 10.1111/jcmm.18497 (PMC11184281; doi:10.1111/jcmm.18497)

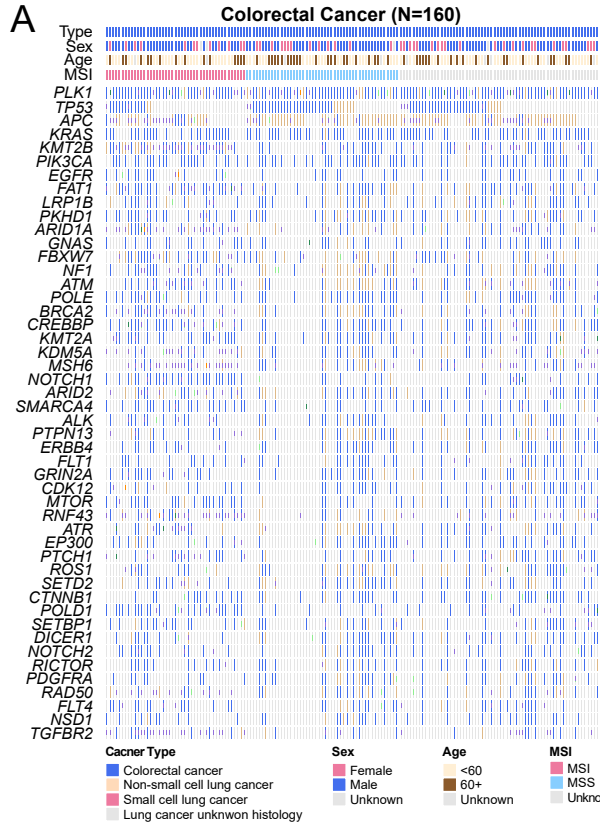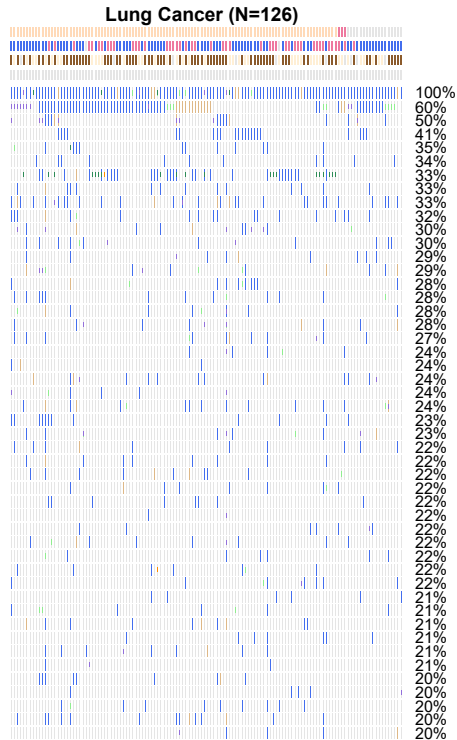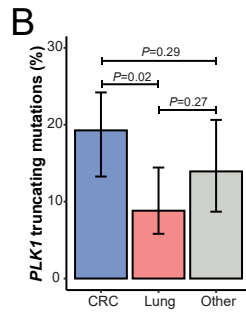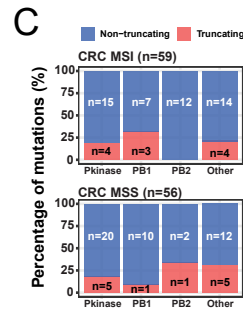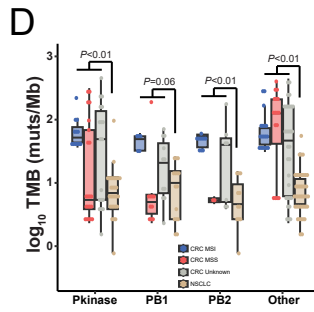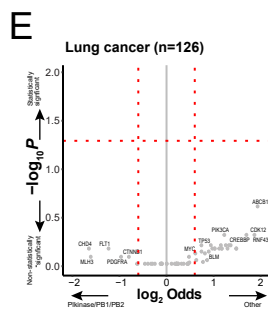

Supplement: Supplementary file 1 — Figure S1. [file JCMM-28-e18497-s001.zip › jcmm18497-sup-0001-Figure S1.pdf]
