# Supplementary material for: Unravelling the role of PLK1 in tumorigenesis by revealing the mutational landscape of colorectal and lung cancer with PLK1 mutations
Source: J Cell Mol Med. 2024 Jun 18;28(12):e18497. doi: 10.1111/jcmm.18497 (PMC11184281; doi:10.1111/jcmm.18497)
Supplement: Supplementary file 4 — Appendix S1. [file JCMM-28-e18497-s003.docx]

**Methods**

***Patients and study design***

The Nanjing Geneseeq Technology Inc. database was queried to identify eligible patients between June 2016 and May 2022, and related medical records were reviewed according to an established protocol of Geneseeq Research Institution. The main inclusion criteria were: 1) adults ≥18 years old; 2) with pathologically confirmed cancer diseases, including lung cancer, colorectal cancer (CRC), breast cancer, hepatobiliary cancer, genitourinary cancer, uterine cancer, etc.; 3) identified with non-synonymous *PLK1* somatic mutations in tumor tissue biopsies. Demographics and clinical characteristics of patients, including age, sex, and cancer staging were also obtained from the Nanjing Geneseeq Technology Inc. database. According to the gene region of *PLK1* mutations, samples were grouped into four subgroups, including the Pkinase subgroup, PB1 subgroup, PB2 subgroup, and the Other subgroup.

From cBioPortal database, a combined CRC dataset (https://bit.ly/43D5zsm) was queried to identify patients harboring *PLK1* alterations, and Microsatellites instability and disease-free survival data were downloaded for data analyses. Also, ten tumor-suppressor genes and ten oncogenes whose prevalence in CRC were approximately 3% were randomly selected, and proportion of truncating mutations of each gene over all its somatic mutations was calculated using cBioPortal data.

***DNA extraction, library preparation, next-generation sequencing, and data processing***

Genomic DNA of tumor tissues was extracted from formalin-fixed, paraffin-embedded (FFPE) samples using QIAamp DNA FFPE Tissue Kit (Qiagen, Dusseldorf, Germany). Peripheral blood (10 mL) was collected and centrifuged (1800x g, 10 minutes, room temperature) within two hours. The plasma fraction was subjected to circulating free DNA extraction using Qiagen QIAamp Circulating Nucleic Acid Kit (Qiagen, Dusseldorf, Germany), followed by purification, qualification (Nanodrop2000, Thermo Fisher Scientific, Waltham, MA, USA), and quantification (dsDNA HS Assay Kit, Life Technologies, Waltham, MA, USA). The KAPA Hyper Prep kit (KAPA Biosystems, Wilmington, MA, USA) was used to prepare sequencing libraries with an optimized manufacturer’s protocol. Customized xGen lockdown probes targeting 425 cancer-relevant genes (GeneseeqPrime™, Nanjing Geneseeq Technology Inc., Nanjing, China) were used for hybridization enrichment, and enriched libraries were sequenced on Illumina sequencing platforms (Illumina, San Diego, CA, USA). Sequencing data were analyzed as previously described ([1](#_ENREF_1)). Single nucleotide variants and indels with the variant allele frequency (VAF) over 0.3% and at least three unique mutant reads were retained. Copy number variations (CNV) with a fold change ≥1.6 and ≤0.6 were identified as CNV amplification and CNV deletion, respectively.

***Statistical analysis***

Descriptive statistics were used for clinical characteristics. Differences in frequencies and medians of independent subgroups were identified using Fisher’s exact test and Wilcoxon signed-rank, respectively. Multivariable linear regression models were fitted to control for the confounding effects. FDR correction was applied for the multiple comparison issue when identifying mutated genes and/or pathway enriched in one subgroup. For each analysis, individuals with missing data were excluded. All quoted *P*-values and FDR-corrected *P*-values were two-tailed, with values <0.05 considered to be statistically significant. Data were analyzed using R software (version 4.0.3), and the *epiR* package.

**Reference**

1. Shu Y, Wu X, Tong X, Wang X, Chang Z, Mao Y, et al. Circulating tumor DNA mutation profiling by targeted next generation sequencing provides guidance for personalized treatments in multiple cancer types. Scientific reports. 2017;7(1):1-11.
